# Micro- and Nanoplastics as Drivers and Modulators of Hepatotoxicity in Zebrafish (*Danio rerio*): Interactions with Environmental Co-Contaminants and Molecular Mechanisms

**DOI:** 10.3390/toxics14060475

**Published:** 2026-05-28

**Authors:** Alexandra Szilagyi, Alexandra Jităreanu, Alina Iliuța Olărița, Carmen Solcan

**Affiliations:** 1 Faculty of Veterinary Medicine, “Ion Ionescu de la Brad” Iasi University of Life Sciences, 8 M. Sadoveanu Alley, 700489 Iasi, Romania; alexandra.szilagyi13@yahoo.com (A.S.); olarita.alinailiuta@yahoo.com (A.I.O.); carmen.solcan@iuls.ro (C.S.); 2 Faculty of Pharmacy, University of Medicine and Pharmacy “Grigore T. Popa”, 700115 Iasi, Romania

**Keywords:** microplastics, nanoplastics, zebrafish, hepatotoxicity, liver histopathology, oxidative stress, metabolic disruption, aquatic toxicology

## Abstract

Micro- and nanoplastics (MNPs) have emerged as pervasive contaminants in aquatic ecosystems, raising concerns regarding their biological impacts on aquatic organisms. The liver plays a central role in metabolism, detoxification, and immune regulation, making it particularly vulnerable to MNP-induced toxicity. Importantly, MNPs also function as vectors and modulators of co-occurring environmental contaminants, including heavy metals, pesticides, antibiotics, PFASs, algal toxins, and polycyclic aromatic hydrocarbons (PAHs), thereby influencing contaminant bioavailability and hepatic toxicity. This narrative review synthesizes current evidence on hepatic alterations induced by micro- and nanoplastic exposure in zebrafish (*Danio rerio*), with emphasis on histopathological changes and underlying mechanisms. Relevant peer-reviewed studies were identified through systematic searches of Web of Science, Scopus, PubMed, and ScienceDirect, covering the period 2013–2026, and screened according to predefined inclusion criteria focusing on hepatic endpoints in zebrafish exposed to micro- and nanoplastics. Across the available literature, MNPs consistently accumulate in hepatic tissue and induce structural alterations, including hepatocellular vacuolization, steatosis, inflammatory infiltration, and necrosis. Mechanistically, these pathological changes are closely linked to oxidative stress, impairment of antioxidant defense systems, reprogramming of lipid and glucose metabolism, and activation of inflammatory and regulated cell death signaling pathways. In addition, interactions with co-occurring environmental contaminants—such as heavy metals, pesticides, antibiotics, and algal toxins—frequently exacerbate hepatic injury through synergistic toxicological mechanisms. Disruption of the gut–liver axis and intestinal microbiota has also emerged as an important contributor to systemic metabolic and inflammatory responses. Overall, zebrafish studies demonstrate that the liver represents a critical target organ for MNP toxicity. Future research should prioritize environmentally realistic exposure scenarios, standardized particle characterization, and integrated multi-omics approaches to improve ecological and human health risk assessment.

## 1. Introduction

Plastic production has increased exponentially over the past few decades, exceeding 400 million tons annually. This has generated large quantities of persistent debris in aquatic ecosystems [1,2]. Larger plastic materials fragment into microplastics (particles < 5 mm) and nanoplastics (typically <1 µm) through physical, chemical, and biological degradation processes. These particles are now widely detected in both marine and freshwater environments worldwide [3]. Micro- and nanoplastics (MNPs) are an emerging class of environmental contaminants. Beyond their direct biological effects, MNPs can also alter the environmental behavior, transport, and bioavailability of co-occurring contaminants through sorption/desorption and leaching processes, thereby contributing to complex mixture toxicity in aquatic organisms. They interact with aquatic organisms at multiple biological levels, raising significant ecological and toxicological concerns.

Fish are particularly susceptible to MNP exposure due to continuous interaction with water, sediments, and food sources. Exposure primarily occurs through ingestion and trophic transfer. Evidence indicates that MNPs move across food webs and may be distributed systemically within organisms [4,5]. Once internalized, MNPs may accumulate in multiple tissues, including the gastrointestinal tract, gills, brain, and liver, with the latter representing a key target organ. Its central role in xenobiotic metabolism and its anatomical connection to the gastrointestinal tract via the portal circulation make it highly vulnerable [6].

The zebrafish (*Danio rerio*) has emerged as a widely used vertebrate model for investigating the biological effects of environmental contaminants, including MNPs. Its suitability for toxicological research is supported by rapid development, optical transparency during early life stages, high fecundity, and substantial genetic homology with mammals [7,8]. In addition, the availability of advanced analytical approaches, including transcriptomics and metabolomics, enables detailed investigation of pollutant-induced molecular mechanisms [9]. These characteristics make zebrafish a powerful model for studying the uptake, biodistribution, and biological effects of MNPs across different developmental stages [5].

Experimental studies demonstrate that MNPs can accumulate in hepatic tissue following exposure via water or diet, where they induce a broad spectrum of toxic effects [6]. These effects include oxidative stress, inflammatory activation, and disruption of lipid and glucose metabolism. These changes are frequently accompanied by structural alterations, including hepatocellular vacuolization, steatosis, and necrosis [10]. Mechanistically, these changes are closely linked to increased reactive oxygen species (ROS) production, impaired antioxidant defenses, and activation of inflammatory and regulated cell death pathways [11,12,13]. In addition to direct particle effects, MNP exposure may also indirectly influence hepatic function by disrupting the intestinal microbiota and the gut–liver axis, thereby amplifying systemic metabolic and inflammatory responses [14].

An important dimension of MNP toxicity involves their interactions with co-occurring environmental contaminants, which are governed by physicochemical processes that determine contaminant release, transfer, and bioavailability. Microplastics can act as both carriers and modulators of chemical exposure for substances such as heavy metals, pesticides, antibiotics, and polycyclic aromatic hydrocarbons (PAHs), thereby altering contaminant bioavailability and toxicity [15,16]. These interactions frequently result in greater oxidative stress, metabolic disruption, and hepatic injury than in single-exposure scenarios [17].

A key conceptual framework for understanding these processes is the dual-origin nature of MNP-associated chemical exposure. MNPs contribute to toxicity through two interconnected mechanisms: (i) the release of intrinsic plastic-associated chemicals (leaching), including additives and residual monomers, and (ii) the adsorption and subsequent transfer of external environmental contaminants (sorption/desorption processes) [18,19]. These mechanisms determine whether MNPs function predominantly as sources of chemical exposure or as carriers that modify contaminant dynamics [20]. Consequently, hepatotoxic effects cannot be interpreted solely as particle-driven phenomena but rather as the result of combined physicochemical and biological interactions.

Critical knowledge gaps persist regarding MNP-induced hepatotoxicity, despite increasing research interest. Variability in particle characteristics, environmental aging, and experimental design complicates direct comparison between studies, while the predominance of simplified experimental systems limits ecological relevance [15,20]. In addition, although researchers have thoroughly documented molecular and biochemical endpoints, fewer studies integrate these findings with detailed histopathological analyses, thereby limiting comprehensive mechanistic understanding [12].

In this context, this narrative review aims to synthesize current evidence on hepatic toxicity induced by micro- and nanoplastics in zebrafish, with particular emphasis on histopathological alterations and underlying molecular mechanisms. By integrating findings across diverse experimental approaches, this review seeks to clarify how MNPs affect liver structure and function and to identify key research priorities to advance aquatic toxicology and environmental health [8,9].

## 2. Materials and Methods

### 2.1. Literature Search Strategy

A structured literature search was conducted to identify peer-reviewed studies investigating hepatic toxicity induced by micro- and nanoplastics (MNPs) in zebrafish (*Danio rerio*). Searches were performed in major scientific databases, including Web of Science, Scopus, PubMed, and ScienceDirect, covering publications from 2013 to 2026, a period corresponding to the rapid expansion of research on plastic pollution and its biological impacts. The search strategy combined multiple keywords related to plastics, zebrafish, and hepatic toxicity, including “microplastics”, “nanoplastics”, “zebrafish”, “*Danio rerio*”, “liver”, “hepatotoxicity”, “oxidative stress”, “metabolism”, “histopathology”, and “toxicology”. Boolean operators (AND, OR) were used to refine searches (e.g., microplastics AND zebrafish AND liver).

**Figure 1:**
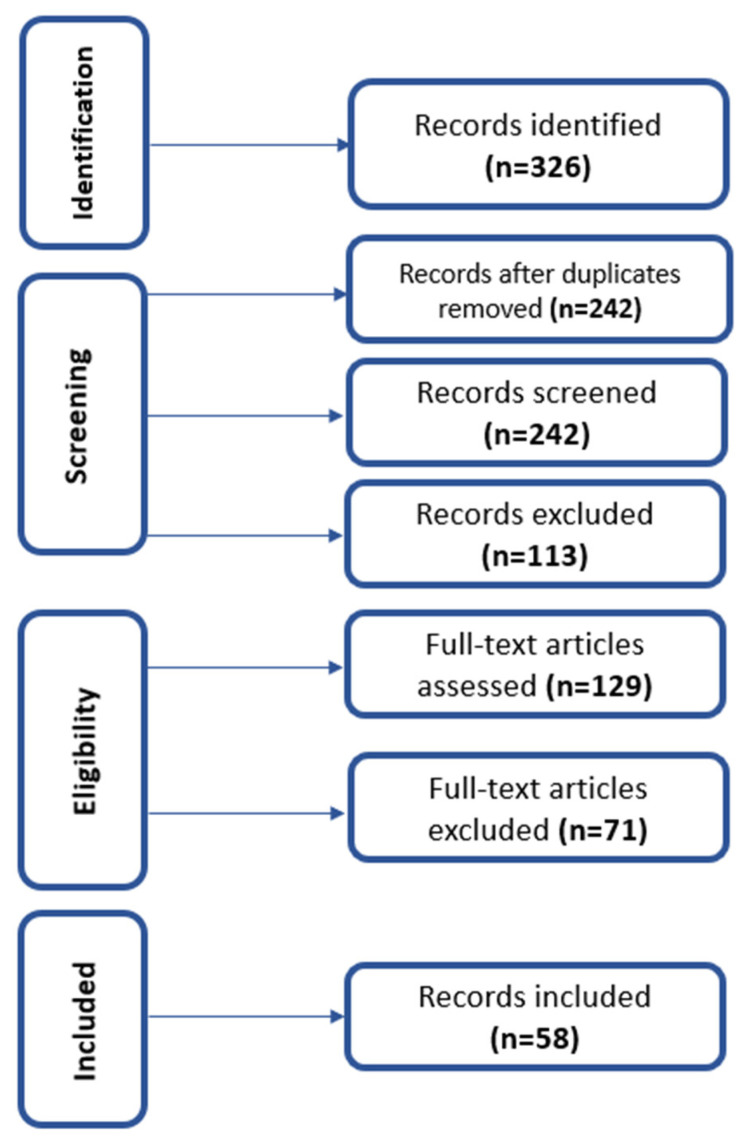
Flow diagram of the literature search and study selection process. Records were identified through database searches and additional sources, screened based on title and abstract, and assessed for eligibility using predefined inclusion and exclusion criteria. A total of 58 studies were included in the final qualitative synthesis.

A total of 326 records were identified through database searches, including additional records identified through reference list screening of relevant articles and reviews. After removal of duplicates, 242 records remained for title and abstract screening. Of these, 113 records were excluded due to lack of relevance to zebrafish models, micro-/nanoplastic exposure, or hepatic/toxicological endpoints. Subsequently, 129 full-text articles were assessed for eligibility. After applying predefined inclusion and exclusion criteria, 71 studies were excluded for lacking hepatic endpoints, using non-zebrafish models, or lacking primary experimental data. Ultimately, 58 studies were included in the qualitative synthesis. Although this review is narrative, the study selection process followed a structured and transparent approach consistent with PRISMA recommendations. The study selection process is summarized in Figure 1.

### 2.2. Study Selection Criteria

Studies were included in the present review if they met the following criteria:

- Experimental investigations involving zebrafish (*Danio rerio*);
- Exposure to microplastics or nanoplastics of any polymer type;
- Evaluation of liver-related outcomes, including histopathology, biochemical markers, molecular responses, or metabolic alterations;
- Publication in peer-reviewed scientific journals.

Studies were excluded if they focused exclusively on environmental monitoring without biological endpoints, investigated non-fish organisms, or examined plastic debris without specifically identifying micro- or nanoplastic particles. Both in vivo experiments and in vitro studies using zebrafish liver cell models were considered, provided that hepatic responses to MNP exposure were investigated.

After applying these criteria, 58 studies were included in the final qualitative synthesis.

### 2.3. Data Extraction and Synthesis

For each study included in the analysis, key information was extracted to facilitate comparison across experiments. The extracted variables included polymer type, particle size and morphology, exposure concentration and duration, zebrafish life stage, and reported hepatic endpoints. Particular attention was given to studies reporting histopathological alterations, including hepatocellular vacuolization, steatosis, necrosis, inflammatory infiltration, and fibrosis-like changes. Importantly, biochemical and molecular endpoints associated with oxidative stress, lipid metabolism, inflammatory signaling, and cell-death pathways were recorded.

Due to substantial variability in experimental design, particle characteristics, and analytical endpoints, a narrative synthesis approach was used rather than quantitative meta-analysis. Evidence was therefore integrated thematically, focusing on major mechanistic pathways and recurring hepatic phenotypes observed across studies. The synthesis was organized into thematic categories, including particle uptake and biodistribution, histopathological alterations, oxidative stress responses, metabolic disruption, immune activation, and interactions with co-pollutants.

This approach enabled the identification of consistent patterns of MNP-induced hepatotoxicity in zebrafish and highlighted methodological differences and knowledge gaps within the current literature. A summary of representative studies included in this review is presented in Table 1.

## 3. Hepatic Uptake, Biodistribution and Toxicokinetics of Micro- and Nanoplastics in Zebrafish

### 3.1. Bioaccumulation of Microplastics and Liver Targeting

Understanding how micro- and nanoplastics (MNPs) reach hepatic tissue is essential for interpreting their downstream toxicological effects. Experimental evidence indicates that MNPs can translocate from primary exposure sites into fish internal organs via trophic transfer and systemic distribution pathways [11,30]. Early studies demonstrated that ingested microplastics can move from the gastrointestinal tract into systemic circulation and accumulate in metabolically active tissues such as the liver and muscle [3]. Importantly, 5 μm polystyrene microplastics were shown to be absorbed from the surrounding water, initially accumulating in the gills and intestine, and were subsequently detected in the liver after prolonged exposure [3]. In contrast, larger particles (20 μm) remained largely confined to the digestive tract, thereby highlighting the critical role of particle size in determining systemic distribution.

Subsequent investigations have confirmed that multiple polymer types—including polyethylene, polypropylene, and polystyrene—can accumulate in zebrafish liver tissue [22,27]. Following entry into the digestive system, these particles may cross epithelial barriers and distribute to internal organs, where hepatic accumulation is frequently associated with oxidative stress, characterized by increased reactive oxygen species (ROS) production and lipid peroxidation [4]. In parallel, disruptions in lipid metabolism, including increased triglyceride accumulation and altered cholesterol levels, suggest that particle deposition contributes directly to liver injury [4]. These observations are consistent with broader experimental evidence demonstrating trophic transfer and systemic redistribution of microplastics in aquatic organisms [10].

Additional evidence from studies on polyethylene terephthalate (PET) fibers and fragments indicates that environmentally relevant particle forms can also reach hepatic tissue [23]. Exposure to PET microfibers has been associated not only with oxidative stress responses but also with endocrine alterations. These include disruption of sex hormone signaling pathways [23]. Environmental factors further modulate these interactions. For example, natural organic matter (NOM) can adsorb onto microplastic surfaces. This process forms an eco-corona that alters physicochemical properties, such as surface charge and hydrophilicity [20]. These modifications may reduce particle aggregation and influence interactions with biological membranes, thereby affecting cellular uptake and bioavailability.

Recent research has also examined biodegradable polymers, revealing that materials such as polylactic acid and polyglycolic acid can similarly accumulate in zebrafish liver tissue [28]. Exposure to these particles has been linked to triglyceride accumulation and dysregulation of genes involved in fatty acid metabolism, indicating that biodegradable plastics are not inherently biologically inert [28]. These findings highlight the importance of evaluating polymer-specific effects in the context of hepatotoxicity.

### 3.2. Biodistribution and Bioaccumulation of Nanoplastics

Compared with microplastics, nanoplastics exhibit a significantly greater capacity to cross epithelial barriers, such as the intestinal epithelium and vascular endothelium, and distribute throughout the organism [11,21]. Their small size and high surface-area-to-volume ratio enhance physicochemical reactivity and promote interactions with cellular membranes and proteins, facilitating uptake and tissue penetration [2]. These properties enhance bioavailability and transfer efficiency across biological systems, including trophic and cellular pathways.

Recent studies employing advanced analytical techniques have quantified nanoplastic distribution across zebrafish tissues [21]. Using MALDI-TOF-MS (Bruker Daltonics, Bremen, Germany), researchers demonstrated that 50 nm polystyrene nanoplastics accumulate in multiple organs, including the intestine, gills, muscle, brain, and liver, with the liver as a major site of retention [21]. Importantly, depuration experiments indicated that nanoplastics persist longer in hepatic tissue than in digestive organs, suggesting slower clearance rates in metabolically active tissues [21].

Particle size strongly influences biodistribution patterns. Comparative studies examining particles ranging from 20 nm to several hundred nanometers have shown that smaller nanoplastics penetrate tissues more deeply and persist longer in internal organs, including the liver and brain [31]. Larger particles tend to remain confined within the gastrointestinal tract, emphasizing the importance of nanoscale dimensions for systemic exposure [11].

Advances in imaging technology have further improved the ability to visualize nanoplastic distribution in living organisms [31]. Label-free hyperspectral stimulated Raman scattering microscopy (Leica Microsystems, Wetzlar, Germany) has enabled three-dimensional visualization of both micro- and nanoplastics within zebrafish tissues, confirming that the intestine and liver are major sites of accumulation following ingestion [31]. These studies also demonstrate that nanoplastics are more likely to reach the circulatory system and secondary organs compared with larger particles [11].

Evidence also suggests that nanoplastics may participate in transgenerational transfer. Experimental work has shown that nanoplastics present in adult zebrafish can be transferred to developing embryos, likely through maternal deposition in oocytes or transport via circulating lipoproteins [26]. This observation aligns with broader findings indicating that nanoplastics can be efficiently transferred across biological compartments and food webs, raising concerns regarding long-term biological effects [32].

### 3.3. Influence of Eco-Corona and Protein Corona Formation

Micro- and nanoplastics rapidly acquire a surface coating—referred to as an eco-corona or protein corona—upon interaction with biological fluids, leading to changes in their physicochemical properties and biological behavior [20]. Evidence from zebrafish liver cell models indicates that corona formation significantly influences particle uptake and cellular responses, with serum-conditioned particles exhibiting altered internalization compared with pristine particles [33]. These findings reflect shifts in uptake pathways and highlight the role of biomolecular coatings in determining particle–cell interactions.

Corona formation is associated with changes in internalization mechanisms, often promoting receptor-mediated endocytosis, including clathrin- and caveolin-dependent pathways, while reducing direct membrane interactions [33]. These alterations can affect intracellular trafficking and downstream responses, including oxidative stress and disruptions in glycolipid metabolism, thereby influencing hepatocellular function.

In addition to modifying cellular interactions, eco-corona formation can influence the sorption and desorption of environmental contaminants, thereby affecting their transport and bioavailability [20]. This has important implications for hepatic exposure, as corona-modified particles may alter the delivery of associated chemicals to the liver. The role of corona formation in shaping contaminant interactions and overall toxicity is further discussed in Section 10.

## 4. Histopathological Alterations in the Liver Induced by Micro- and Nanoplastics

Histopathological examination remains a fundamental approach for assessing hepatotoxicity in aquatic toxicology, providing direct evidence of tissue injury. In zebrafish (*Danio rerio*), exposure to micro- and nanoplastics (MNPs) has consistently been associated with structural alterations in hepatic tissue, including hepatocellular vacuolization, lipid accumulation, inflammatory infiltration, necrosis, and sinusoidal congestion [13,27]. These changes reflect disruption of hepatic architecture and metabolic homeostasis and are widely reported across experimental exposure models [4].

Emerging evidence indicates that these histopathological alterations may be modulated by interactions between MNPs and co-occurring environmental contaminants, as particles can influence local exposure profiles through sorption/desorption processes [15,20]. This interaction contributes to variability in tissue-level responses under different experimental and environmental conditions and may partially explain discrepancies between studies.

Current evidence suggests that many of these alterations are driven primarily by direct particle effects, particularly through intracellular accumulation and physical interactions with hepatocytes [3,11]. Chemical-mediated contributions may also influence these responses under environmentally relevant conditions [19].

### 4.1. Hepatocellular Vacuolization and Steatosis

Hepatocellular vacuolization is among the most frequently reported hepatic alterations following MNP exposure, often accompanied by lipid droplet accumulation indicative of steatosis. Early investigations demonstrated that zebrafish exposed to polystyrene microplastics exhibited significant lipid accumulation in hepatocytes, reflecting disruption of lipid metabolism and impaired hepatic function [3,4]. These lipid droplets are typically observed as clear cytoplasmic vacuoles that displace the nucleus toward the cell periphery, a hallmark of fatty liver degeneration.

Building on these initial findings, subsequent studies have confirmed that exposure to multiple polymer types, including polyethylene and polypropylene, induces similar morphological changes in zebrafish liver tissue [22,27]. Histological analyses frequently reveal hepatocyte swelling, cytoplasmic vacuolation, and disorganization of hepatic cords, often accompanied by increased triglyceride levels and altered expression of genes regulating lipid metabolism [4]. These findings support a consistent link between microplastic exposure and the development of steatosis-like phenotypes.

Nanoplastics appear to exert more pronounced effects on hepatic lipid metabolism due to their enhanced cellular uptake and intracellular interactions [5,11]. Exposure to polystyrene nanoplastics has been shown to induce extensive lipid droplet accumulation and dysregulation of genes involved in fatty acid synthesis and β-oxidation, changes consistent with early stages of non-alcoholic fatty liver disease (NAFLD)-like hepatic alterations [34]. These alterations suggest that nanoplastic exposure may contribute to progressive metabolic dysfunction and impaired hepatic homeostasis.

Furthermore, co-delivery of lipophilic contaminants associated with particle surfaces may further exacerbate lipid accumulation and metabolic imbalance, particularly under environmentally relevant exposure conditions [15,19].

### 4.2. Hepatocellular Necrosis and Apoptosis

In addition to lipid accumulation, several studies have reported hepatocellular necrosis and apoptosis following MNP exposure [13,27]. Necrotic lesions typically present as localized areas of cell death characterized by nuclear pyknosis, karyorrhexis, and cytoplasmic degeneration, indicating irreversible hepatocyte injury and disruption of tissue integrity [3,24].

Apoptosis has also been thoroughly documented in zebrafish liver exposed to micro- and nanoplastics, with molecular analyses revealing activation of caspase-dependent signaling pathways and upregulation of pro-apoptotic genes [35]. Histological observations frequently include nuclear condensation and chromatin fragmentation, consistent with programmed cell death in hepatocytes.

The coexistence of necrosis and apoptosis suggests that MNP-induced hepatotoxicity involves multiple cellular injury pathways, with oxidative stress and mitochondrial dysfunction acting as key upstream triggers [6]. These mechanisms contribute to hepatocyte loss and impaired liver function.

These processes may be further intensified in the presence of particle-associated contaminants, which can increase intracellular toxic burden through vector-mediated delivery and amplify cellular damage [15,20].

### 4.3. Inflammatory Responses and Immune Cell Infiltration

Inflammation is a common histopathological feature observed in the zebrafish liver following MNP exposure, often characterized by immune cell infiltration, sinusoidal dilation, and vascular congestion [13,27]. These changes indicate activation of innate immune pathways and contribute to the progression of hepatic injury.

At the molecular level, MNP exposure is associated with increased expression of pro-inflammatory cytokines and activation of signaling pathways, such as NF-κB and MAPK, which regulate immune responses and inflammatory processes [6,17]. Activation of these pathways promotes recruitment of immune cells and amplifies local inflammatory responses, exacerbating hepatocellular damage.

Chronic inflammation may also contribute to early fibrotic changes, including increased extracellular matrix deposition and altered expression of fibrosis-related genes [27]. While advanced fibrosis is rarely observed in zebrafish liver during short-term experimental exposures, these findings highlight the potential for progressive liver pathology with prolonged or repeated MNP exposure.

**Figure 2:**
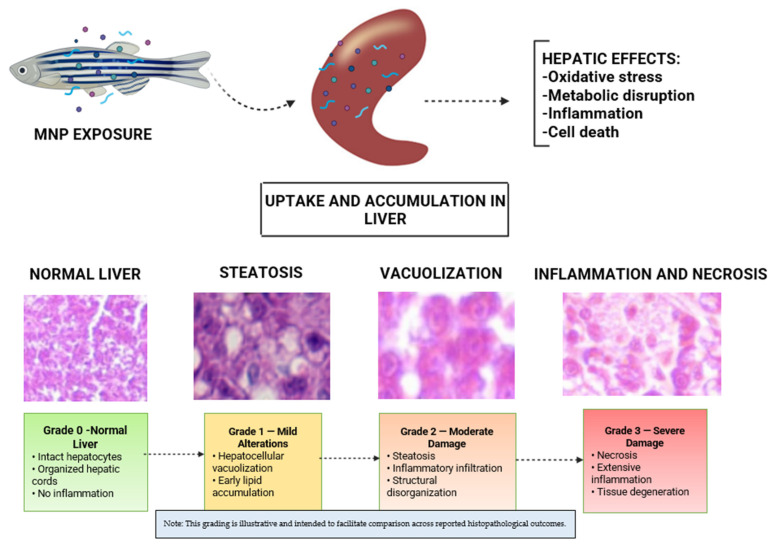
Representative histopathological alterations induced by micro- and nanoplastics in zebrafish liver. Schematics and selected elements are adapted from [36,37,38] with modifications. Created in part using BioRender (BioRender Inc., Toronto, ON, Canada).

Representative histopathological alterations induced by micro- and nanoplastics in zebrafish liver are summarized in Figure 2.

## 5. Oxidative Stress and Antioxidant Responses in Zebrafish Liver

Oxidative stress is a central and consistently reported mechanism underlying MNP-induced hepatotoxicity in zebrafish, characterized by increased reactive oxygen species (ROS) production, lipid peroxidation, and disruption of antioxidant defenses [4,17]. These biochemical disturbances often occur alongside histopathological alterations, indicating that oxidative stress is a key driver of liver injury [13]. This response is primarily attributed to direct particle effects, including surface reactivity and intracellular accumulation. However, interactions with co-occurring contaminants may further modulate oxidative responses under environmentally relevant conditions [15]. The biological effects of microplastics are mediated through multiple mechanisms, including oxidative stress, inflammation, and physical interactions with tissues [39]. While oxidative stress is frequently attributed to direct particle–cell interactions, contributions from leached additives and sorbed co-contaminants should also be considered, particularly under environmentally realistic exposure conditions. This is especially relevant under environmentally realistic exposure conditions [20].

### 5.1. Reactive Oxygen Species Generation and Lipid Peroxidation

Exposure to micro- and nanoplastics has been repeatedly shown to elevate intracellular ROS levels in zebrafish liver [4,5]. These reactive molecules include superoxide anions, hydroxyl radicals, and hydrogen peroxide. When produced in excess, they can damage proteins, lipids, and nucleic acids. Experimental studies involving polystyrene and polyethylene particles have demonstrated significant increases in ROS production, often in a dose-dependent manner, supporting a direct link between particle exposure and oxidative imbalance [22].

Elevated ROS levels frequently lead to lipid peroxidation of cellular membranes, a process commonly assessed through increased malondialdehyde (MDA) concentrations [13,17]. Increased hepatic MDA levels indicate oxidative degradation of polyunsaturated fatty acids and loss of membrane integrity, contributing to hepatocellular dysfunction. These findings highlight lipid peroxidation as a key downstream consequence of ROS overproduction.

Nanoplastics appear to induce more pronounced oxidative effects than larger particles. This is due to their enhanced cellular uptake and intracellular interactions [5,11]. Their small size facilitates penetration into subcellular compartments, including mitochondria. Inside these organelles, nanoplastics can disrupt electron transport processes and increase ROS generation. Comparative studies demonstrate higher levels of oxidative damage following nanoplastic exposure, underscoring the importance of particle size in determining toxicity.

Environmental stressors can further amplify oxidative responses. Co-exposure to contaminants such as pharmaceuticals or heavy metals has been shown to increase ROS production and lipid peroxidation beyond the levels observed under single-exposure conditions [15,17]. These findings suggest that oxidative stress is not only particle-driven but also influenced by combined environmental factors that enhance cellular vulnerability.

In parallel with increased ROS production, MNP exposure induces characteristic alterations in antioxidant defense systems [4,13]. Early exposure is often associated with compensatory increases in antioxidant enzymes such as superoxide dismutase (SOD) and catalase (CAT), whereas prolonged or higher-dose exposure leads to depletion of these defenses. Disruption of glutathione-related systems, including glutathione peroxidase (GPx) and reduced glutathione (GSH), further impairs redox homeostasis and promotes oxidative damage.

While many studies attribute ROS generation to particle-induced cellular stress, particularly mitochondrial dysfunction, additional contributions from chemical exposure—such as leached additives or adsorbed pollutants—may also enhance oxidative responses, particularly under co-exposure conditions.

### 5.2. Disruption of Antioxidant Defense Systems

Exposure to microplastics has been shown to disrupt these protective mechanisms in the zebrafish liver. Consistent with the patterns described above, several studies report decreased SOD and CAT activity following plastic exposure, suggesting that antioxidant defenses may become overwhelmed by excessive ROS production [4]. Across studies, antioxidant enzyme responses (e.g., SOD, CAT) often exhibit a biphasic pattern, with increases of approximately 20–80% during early exposure followed by significant depletion under prolonged or higher-dose conditions, depending on particle characteristics and exposure parameters. Reduced antioxidant activity can exacerbate oxidative damage and contribute to the structural liver alterations observed in histopathological analyses.

For example, short-term exposure to polyethylene microplastics (typically ranging from several days up to 1–2 weeks) has been associated with increased GST activity, indicating an adaptive response to detoxify reactive intermediates. Nevertheless, prolonged exposure (generally extending beyond 2–4 weeks) often leads to depletion of antioxidant reserves and progressive oxidative damage [13,22].

Alterations in glutathione metabolism represent another important indicator of oxidative stress [4,13]. Reduced glutathione (GSH) is a major intracellular antioxidant that neutralizes reactive oxygen species. Several investigations have reported decreased hepatic GSH levels in zebrafish exposed to micro- and nanoplastics, reflecting impaired redox regulation and increased oxidative burden.

### 5.3. Molecular Pathways Associated with Oxidative Stress

At the molecular level, oxidative stress triggered by MNP exposure is closely linked to activation of several intracellular signaling pathways. Among the most frequently implicated pathways are the mitogen-activated protein kinase (MAPK) and nuclear factor-κB (NF-κB) signaling cascades, both of which regulate inflammatory responses and cellular stress reactions [6,17].

Activation of MAPK signaling pathways (including ERK, JNK, and p38) has been observed in zebrafish following exposure to various microplastic particles. Recent single-cell transcriptomic analyses further demonstrate that nanoplastic exposure induces heterogeneous activation of oxidative stress–related and inflammatory pathways across distinct hepatic cell populations, highlighting cell-type-specific susceptibility and signaling responses [6]. These pathways regulate cellular responses to oxidative stress and can promote apoptosis or inflammatory signaling when activated under pathological conditions [40].

Similarly, NF-κB activation has been associated with increased expression of pro-inflammatory cytokines and immune mediators in plastic-exposed zebrafish [17]. This signaling cascade links oxidative stress to inflammation and contributes to the amplification of hepatic injury. The interplay between oxidative and inflammatory pathways represents a key feature of MNP-induced toxicity.

In addition to the above signaling pathways, mitochondrial dysfunction also appears to contribute to oxidative toxicity. Plastic particles entering hepatocytes may interfere with mitochondrial respiration, leading to leakage of electrons from the electron transport chain and increased ROS generation. This mitochondrial stress can initiate apoptotic signaling pathways and further aggravate hepatic damage [5].

Taken together, these findings indicate that oxidative stress arises from the interplay between ROS overproduction, disruption of antioxidant defenses, and activation of stress-responsive signaling pathways, ultimately driving hepatocellular damage [4,6]. This integrative mechanism underlies the downstream metabolic and inflammatory alterations associated with MNP exposure and is further amplified under co-exposure conditions, as discussed in Section 10. Under environmentally realistic conditions, oxidative responses are likely shaped by both direct particle effects and chemical-mediated mechanisms associated with co-occurring contaminants.

## 6. Metabolic Reprogramming of Hepatic Lipid and Glucose Pathways

The liver plays a central role in lipid and glucose regulation, and disruption of these pathways represents one of the most consistent hepatic consequences of micro- and nanoplastic (MNP) exposure in zebrafish (*Danio rerio*) [4,5]. Across experimental studies, MNPs have been shown to alter biochemical parameters, gene expression profiles, and lipid metabolism, often in parallel with steatosis and hepatocellular vacuolization [41]. These findings support metabolic reprogramming as a key mechanism linking particle exposure to structural liver injury and are closely linked to the oxidative stress responses described in Section 5. These metabolic alterations may arise from both direct particle-induced stress and chemical-mediated effects associated with co-transported contaminants.

### 6.1. Microplastics and Hepatic Glycolipid Metabolism

Early studies demonstrated that exposure to polystyrene microplastics disrupts hepatic lipid metabolism, leading to lipid accumulation and altered expression of genes involved in fatty acid synthesis and β-oxidation [24]. Similar effects have been reported for other polymer types, including polyethylene and polypropylene, which are associated with increased hepatic triglyceride levels and altered cholesterol homeostasis [22]. These alterations are linked to dysregulation of key metabolic regulators such as sterol regulatory element-binding proteins (SREBPs) and peroxisome proliferator-activated receptors (PPARs), which play essential roles in maintaining lipid balance.

In addition to lipid metabolism, MNP exposure affects glucose homeostasis by modulating pathways involved in glycolysis and gluconeogenesis [14,34]. Disruption of these processes reflects broader disturbances in hepatic energy metabolism and contributes to metabolic imbalance. Evidence supports that micro- and nanoplastics impair glycolipid homeostasis and promote steatosis consistent with fatty liver–like phenotypes in zebrafish.

### 6.2. Nanoplastic-Induced Lipidomic Alterations

Lipidomic analyses have revealed significant alterations in phospholipids, sphingolipids, and neutral lipids in zebrafish liver following nanoplastic exposure [41], including increased triglyceride levels, altered phosphatidylcholine/phosphatidylethanolamine balance, and accumulation of ceramide-related lipids. These changes indicate not only enhanced lipid storage but also disruption of membrane composition and lipid-mediated signaling pathways, suggesting a broader reprogramming of hepatic metabolic homeostasis.

Studies using zebrafish liver cell models further demonstrate that micro- and nanoplastics can alter key lipid classes, including phosphatidylcholine, phosphatidylethanolamine, ceramides, and sphingolipids [26]. These alterations may affect membrane integrity, intracellular signaling, and cellular stress responses. In parallel, metabolomic analyses indicate broader disruptions in amino acid metabolism, energy production, and fatty acid pathways, supporting the involvement of multiple interconnected metabolic networks [40].

### 6.3. Interaction Between Diet and Microplastic Exposure

Dietary status represents an important modifier of MNP-induced metabolic effects. In zebrafish exposed to high-fat diets, co-exposure to polystyrene microplastics has been shown to exacerbate hepatic lipid accumulation, steatosis, and dysregulation of lipid metabolism genes compared with either stressor alone [42]. These findings indicate that nutritional stress can amplify susceptibility to MNP-induced metabolic dysfunction and highlight the importance of considering combined environmental and physiological stressors.

Biodegradable polymers such as polylactic acid (PLA) and polyglycolic acid (PGA) have also been associated with lipid accumulation and altered expression of metabolic genes [28]. These results challenge the assumption that biodegradable materials are inherently less toxic and suggest that polymer degradation does not eliminate biological effects. Together, these findings emphasize that both particle characteristics and host metabolic state influence the extent of hepatic metabolic reprogramming. These metabolic disturbances may therefore reflect the combined effects of particle exposure and contaminant-mediated toxicity under mixture exposure scenarios.

## 7. Immune and Inflammatory Responses in the Zebrafish Liver

The liver is a central organ in innate immune regulation, and exposure to micro- and nanoplastics (MNPs) can trigger inflammatory responses within hepatic tissue. In zebrafish, these responses are characterized by cytokine induction, immune cell recruitment, and activation of intracellular signaling pathways associated with inflammation. In addition to direct particle effects, inflammatory responses may be modulated by interactions with co-occurring contaminants, as MNPs can influence the bioavailability and hepatic delivery of pro-inflammatory compounds [15]. Accordingly, inflammatory responses should be interpreted within a dual-origin framework, reflecting both particle-driven effects and chemical-mediated signaling associated with contaminants.

### 7.1. Activation of Inflammatory Signaling Pathways

Microplastic exposure has been consistently associated with activation of key inflammatory signaling pathways, particularly nuclear factor-κB (NF-κB) and mitogen-activated protein kinase (MAPK) cascades [6,17]. These pathways play central roles in cellular stress responses and regulate the transcription of pro-inflammatory mediators, including cytokines and chemokines.

In zebrafish exposed to polystyrene microplastics, increased expression of pro-inflammatory cytokines, including TNF-α, IL-1β, and IL-6, has been reported. Nanoplastics may induce more pronounced inflammatory responses due to their enhanced cellular uptake and intracellular interactions [11]. These findings indicate that inflammatory signaling represents a downstream consequence of oxidative stress and metabolic disruption induced by MNP exposure.

### 7.2. Immune Cell Recruitment and Hepatic Inflammation

Histopathological studies frequently demonstrate inflammatory cell infiltration, sinusoidal dilation, and vascular congestion in the zebrafish liver following MNP exposure [13,27]. These features indicate local immune activation and are consistent with the structural alterations described in Section 4. The presence of infiltrating immune cells suggests activation of innate immune responses and progression toward tissue-level inflammation.

Transcriptomic analyses further support these findings, showing altered expression of genes involved in immune regulation, antigen presentation, and inflammatory signaling pathways related to NF-κB/MAPK signaling, cytokine-mediated responses, and antigen processing [40]. These molecular changes, together with histological evidence, indicate that inflammation is a major component of MNP-induced hepatotoxicity and contributes to the progression of liver injury.

### 7.3. Interactions Between Metabolic Disturbance and Inflammation

Metabolic disruption and inflammation are closely interconnected processes in MNP-induced hepatotoxicity [6]. Lipid accumulation within hepatocytes can promote oxidative stress and activate inflammatory signaling pathways, while pro-inflammatory cytokines such as TNF-α and IL-6 can impair insulin signaling and further disrupt lipid metabolism. This reciprocal relationship suggests that metabolic and immune responses are functionally linked rather than independent processes.

Oxidative stress acts as a key upstream trigger in this interaction, with increased ROS production promoting activation of the NF-κB and MAPK pathways [17]. These pathways amplify cytokine-mediated responses, contributing to sustained hepatic inflammation and tissue damage. Together, these findings support an integrated model in which oxidative stress, metabolic dysregulation, and inflammation form a self-reinforcing cycle that drives hepatotoxicity. Consequently, inflammatory responses should be interpreted within the broader context of mixed contaminant exposure and MNP-mediated modulation of chemical bioavailability.

## 8. Cell Death Pathways: Apoptosis, Ferroptosis, and Other Regulated Cell Death Mechanisms

Micro- and nanoplastic (MNP)-induced hepatic injury in zebrafish can progress from adaptive cellular stress responses to hepatocyte death through multiple interconnected mechanisms. These processes are primarily driven by oxidative stress, mitochondrial dysfunction, and inflammatory signaling, which together determine the extent of cellular damage. Interactions with co-occurring contaminants may further amplify regulated cell death signaling pathways [15].

These mechanisms are not isolated but arise from the cumulative effects of oxidative imbalance, metabolic disruption, and inflammation described in previous sections. As a result, hepatocyte death represents a downstream convergence point of multiple stress-response pathways and plays a key role in translating molecular alterations into histopathological damage [4].

### 8.1. Apoptotic Pathways

Apoptosis is the most consistently reported form of cell death following MNP exposure in zebrafish liver [35]. It is characterized by nuclear condensation, chromatin fragmentation, and activation of caspase-dependent pathways. Molecular analyses frequently reveal upregulation of pro-apoptotic markers such as Bax, caspase-3, and caspase-9, together with downregulation of anti-apoptotic Bcl-2, indicating activation of programmed cell death mechanisms [6,35].

These alterations reflect activation of the intrinsic (mitochondrial) apoptotic pathway, in which oxidative stress-induced mitochondrial damage leads to cytochrome c release and downstream caspase activation [5]. This pathway appears to represent a primary cellular response to moderate stress, enabling the controlled removal of damaged hepatocytes while maintaining tissue integrity.

### 8.2. Ferroptosis and Oxidative Cell Death Mechanisms

In addition to apoptosis, ferroptosis has emerged as a relevant contributor to MNP-induced hepatotoxicity [35,41]. Ferroptosis is an iron-dependent form of regulated cell death driven by lipid peroxidation and disruption of redox homeostasis. Experimental studies report increased lipid peroxidation and altered expression of genes involved in iron metabolism and antioxidant regulation following nanoplastic exposure.

Although direct evidence in adult zebrafish liver remains limited, the observed accumulation of lipid peroxidation products and depletion of antioxidant defenses strongly support the involvement of ferroptosis under conditions of severe oxidative stress [17]. Compared with apoptosis, ferroptosis appears more closely associated with sustained oxidative imbalance and membrane lipid damage, linking redox dysregulation to irreversible cellular injury.

### 8.3. Integrated and Emerging Cell Death Pathways

Beyond apoptosis and ferroptosis, additional regulated cell death mechanisms may contribute to MNP-induced toxicity. Recent studies have proposed the involvement of PANoptosis, a coordinated pathway integrating features of apoptosis, pyroptosis, and necroptosis, particularly under conditions of combined metabolic and inflammatory stress [43]. This emerging concept reflects the integration of multiple signaling pathways activated simultaneously in response to complex environmental exposures.

The coexistence of multiple cell death pathways highlights the complexity of MNP-induced hepatotoxicity. Apoptosis may predominate during early or moderate stress responses, whereas ferroptosis and inflammation-associated mechanisms become more relevant under sustained oxidative and inflammatory conditions [6]. This coordinated activation provides a mechanistic link between molecular disturbances and the histopathological alterations described in Section 4. The activation of regulated cell death pathways may therefore arise from the combined influence of particle-induced stress and co-delivered environmental contaminants.

**Figure 3:**
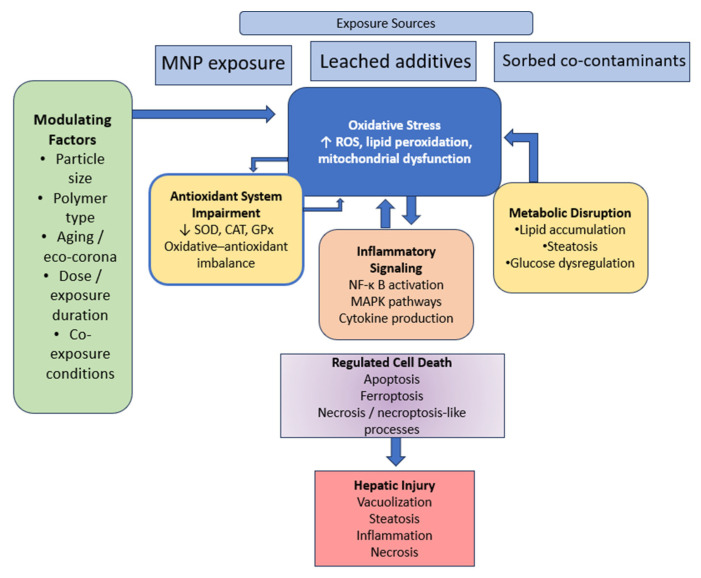
Integrated mechanistic model of micro- and nanoplastic (MNP)-induced hepatotoxicity in zebrafish (*Danio rerio*). The diagram illustrates the interconnections among oxidative stress, metabolic disruption, inflammatory signaling, and regulated cell death pathways. MNP exposure, together with leached additives and sorbed co-contaminants, induces oxidative stress, which acts as a central hub linking mitochondrial dysfunction, lipid peroxidation, and downstream signaling cascades. Bidirectional interactions between oxidative stress and inflammatory pathways, as well as feedback loops involving metabolic disruption and impairment of the antioxidant system, contribute to the amplification of cellular damage. These processes culminate in hepatocellular injury, including steatosis, inflammation, and necrosis. The model also highlights the modulatory role of particle properties, exposure conditions, and co-exposure scenarios. Arrows indicate mechanistic interactions and progression between pathways, while bidirectional arrows represent reciprocal feedback mechanisms.

These interrelated pathways form a convergent network in which oxidative stress, metabolic disruption, and inflammatory signaling collectively drive hepatocyte death. This integrated framework is summarized in Figure 3, which illustrates the interconnected mechanisms underlying MNP-induced hepatotoxicity.

## 9. Gut–Liver Axis and Systemic Effects of Micro- and Nanoplastics

The gastrointestinal tract serves as a primary route of entry for micro- and nanoplastics (MNPs) in aquatic organisms, and intestinal exposure can significantly influence hepatic function via the gut–liver axis [44]. This bidirectional axis integrates epithelial barrier integrity, microbial composition, inflammatory signaling, and hepatic metabolism, providing a mechanistic link between local intestinal effects and systemic toxicity. Disruption of this axis has emerged as a key pathway through which MNP exposure contributes to liver injury.

### 9.1. Intestinal Barrier Disruption, Microbiota Dysbiosis, and Hepatic Consequences

MNP exposure can induce structural and functional alterations in the zebrafish intestine, including epithelial damage, altered mucus production, and changes in goblet cell density [27,44]. These changes increase intestinal permeability and facilitate the translocation of particles, microbial products, and inflammatory mediators into systemic circulation. Through portal transport, these gut-derived signals reach the liver, where they contribute to inflammation and metabolic dysregulation. Nanoplastics may be particularly relevant in this context due to their enhanced ability to cross epithelial barriers [11].

In parallel, MNP exposure alters the composition and diversity of the intestinal microbiota, often characterized by reductions in beneficial taxa and enrichment of opportunistic or potentially pathogenic bacteria [26]. Functionally, this dysbiosis can impair key microbial processes such as short-chain fatty acid production and disrupt bile acid metabolism, both of which are essential for maintaining intestinal barrier integrity and regulating metabolism. Increased abundance of opportunistic bacteria may also elevate endotoxin levels, particularly lipopolysaccharides (LPS), thereby promoting inflammatory signaling.

Together, intestinal barrier disruption and microbiota dysbiosis contribute to a coordinated cascade linking gut and liver pathology [14]. Increased permeability facilitates the translocation of microbial products and altered metabolites into the portal circulation, while dysbiosis modifies the production of bioactive compounds that regulate host metabolism and immune responses. The combined influx of inflammatory mediators and altered metabolites to the liver promotes oxidative stress, immune activation, and metabolic dysfunction, ultimately contributing to hepatocellular injury [14].

MNPs may influence gut–liver signaling not only through epithelial disruption but also by modifying the transport and bioavailability of associated compounds, thereby shaping systemic exposure patterns.

### 9.2. Systemic Signaling and the Gut–Liver–Brain Axis

Gut-derived inflammatory mediators and metabolic signals can further influence hepatic gene expression and function, supporting a systemic integration of responses to MNP exposure [40,41]. Transcriptomic analyses reveal coordinated alterations in pathways related to metabolism, immune regulation, and oxidative stress across both intestinal and hepatic tissues. These findings reinforce the mechanistic link between intestinal disruption and liver injury.

Beyond the gut–liver axis, MNP-induced intestinal alterations may also impact broader systemic pathways, including the gut–liver–brain axis [28]. Evidence from zebrafish suggests that intestinal and hepatic changes may be associated with neurobehavioral effects mediated by metabolic and inflammatory signaling between organs. Although this area remains less well characterized, it highlights the potential for MNP exposure to affect multiple organ systems simultaneously.

Overall, the gut–liver axis provides a unifying framework for understanding how intestinal exposure to MNPs contributes to hepatic injury [14,44]. Rather than acting independently, barrier disruption, microbiota dysbiosis, inflammation, and metabolic alterations form an interconnected cascade that drives systemic toxicity and amplifies liver damage.

## 10. Micro- and Nanoplastics as Dual-Origin Drivers of Mixed Contaminant Hepatotoxicity

In aquatic environments, micro- and nanoplastics (MNPs) rarely occur in isolation; they often coexist with contaminants such as heavy metals, pesticides, pharmaceuticals, polycyclic aromatic hydrocarbons (PAHs), per- and polyfluoroalkyl substances (PFASs), and algal toxins [16]. These components participate in complex transport and transfer processes across aquatic food webs and biological systems, influencing exposure pathways and toxicological outcomes. This section summarizes how MNPs interact with co-occurring contaminants through physicochemical and biological mechanisms, with emphasis on hepatic consequences in zebrafish.

### 10.1. Physicochemical Mechanisms of MNP–Contaminant Interactions

In aquatic systems, interactions between MNPs and contaminants are governed by physicochemical processes that influence contaminant fate, bioavailability, and toxicity [8]. These interactions determine whether plastics act as sources, carriers, or modulators of chemical exposure across biological systems.

MNPs contribute to chemical exposure through two primary mechanisms: (i) release of intrinsic plastic-associated chemicals (leaching) and (ii) adsorption of external pollutants (sorption) [45]. Plastic additives—including plasticizers, stabilizers, flame retardants, pigments, and residual monomers—can leach from polymer matrices, particularly under environmental weathering conditions such as UV radiation, oxidation, and mechanical abrasion. Smaller particles may enhance leaching due to their higher surface-area-to-volume ratio, increasing chemical release into aquatic systems.

Simultaneously, MNPs can adsorb a wide range of environmental contaminants through hydrophobic partitioning, electrostatic interactions, and π–π interactions [7]. These processes are influenced by polymer type, particle size, surface aging, pH, salinity, temperature, and dissolved organic matter. Surface modifications, including eco-corona formation, further alter particle properties and influence contaminant binding, cellular uptake, and toxicokinetics [33].

Following ingestion, desorption becomes a key determinant of internal exposure [19,20]. Gastrointestinal conditions—including pH shifts, enzymatic activity, and bile salts—facilitate the release of both intrinsic additives and adsorbed contaminants. In zebrafish, this process is closely linked to the gut–liver axis, where released compounds are transported to the liver for metabolism and detoxification [14].

Depending on environmental conditions and binding affinities, MNPs may act as either vectors (enhancing contaminant uptake) or sinks (reducing bioavailability) [15]. The resulting toxicological impact reflects a dynamic balance between these roles rather than a single dominant mechanism.

### 10.2. Combined Biological Impacts in Zebrafish

Co-exposure studies demonstrate that micro- and nanoplastics (MNPs) can significantly modify the toxicity of environmental contaminants in zebrafish. However, these effects are highly context-dependent [15,16]. Depending on contaminant type, particle characteristics, and exposure conditions, MNPs may either enhance or mitigate toxic effects. Biological factors such as age and physiological status further influence susceptibility, with older zebrafish often exhibiting more pronounced histopathological damage [25].

Heavy metals represent a well-characterized class of co-contaminants. Interactions between MNPs and metals such as cadmium have been shown to increase hepatic accumulation and exacerbate oxidative stress and metabolic disruption [46,47,48]. Similarly, co-exposure to MNPs and fluorinated compounds, including PFOS and related substances, has been associated with enhanced lipid metabolic disturbances and hepatotoxicity [49]. These findings indicate that MNPs can alter the bioavailability of metal and persistent pollutants, thereby intensifying liver injury.

Organic pollutants and pesticides also exhibit modified toxicity in the presence of MNPs. Interactions with compounds such as chlorpyrifos and imidacloprid have been linked to increased oxidative stress, altered gene expression, and microbiota-mediated effects [45,50]. In addition, co-exposure to polycyclic aromatic hydrocarbons (e.g., phenanthrene or benzo[a]pyrene) can enhance pollutant uptake and amplify oxidative damage through trophic transfer mechanisms [51].

Pharmaceuticals and emerging contaminants further illustrate the complexity of mixture toxicity. For example, antibiotics such as oxytetracycline and sulfonamides have been shown to induce dysbiosis, inflammatory responses, and secondary hepatic effects when combined with microplastics [52]. Likewise, co-exposure to nanoplastics and algal toxins such as microcystin-LR increases toxin bioavailability and aggravates oxidative liver damage [43,53]. Additional studies indicate that inorganic compounds such as sodium fluoride may also exhibit enhanced metabolic toxicity when combined with nanoplastics [54].

Evidence also suggests that MNPs can influence contaminant toxicokinetics through vector-mediated transport and altered bioaccumulation. For example, microplastics have been shown to increase cadmium uptake and retention in aquatic organisms, contributing to higher internal exposure levels [55]. These interactions highlight the role of MNPs as modulators of contaminant distribution within biological systems.

Physiological factors may further modulate these combined effects. For instance, co-exposure to MNPs and high-fat diets has been shown to exacerbate hepatic steatosis and metabolic dysregulation, indicating that nutritional status can influence susceptibility to mixture toxicity [42]. In addition, environmentally aged particles may exhibit altered sorption capacity and surface reactivity, leading to changes in contaminant binding affinity, bioavailability, and cellular uptake, which can in turn modify oxidative stress responses and overall toxicity outcomes [8].

Overall, these findings demonstrate that MNP–contaminant interactions produce complex and often synergistic effects that amplify oxidative stress, metabolic disruption, and inflammatory responses in zebrafish liver. These combined effects are consistent with the histopathological alterations described in Section 4, including increased steatosis, hepatocellular degeneration, and inflammatory infiltration under co-exposure conditions. Key interactions between MNPs and co-contaminants are summarized in Table 2.

### 10.3. Hepatic Implications of Combined Microplastic and Co-Contaminant Exposure

The liver represents a primary target organ for combined exposure to MNPs and environmental contaminants due to its central role in xenobiotic metabolism and its anatomical connection to the gastrointestinal tract [3,14]. As a result, both particle-associated and dissolved contaminants converge at the hepatic level, increasing overall toxic burden. Similar mechanisms have been described in mammalian systems, supporting the conservation of oxidative stress and inflammation pathways [5].

Under co-exposure conditions, biological responses are not independent. Rather, they are interconnected and often amplified [15,17]. Enhanced contaminant delivery and altered bioavailability intensify cellular stress responses, promoting metabolic disturbances, including lipid accumulation and impaired glucose regulation, ultimately contributing to steatosis [4].

In parallel, co-exposure enhances inflammatory signaling and cell death pathways [6]. Activation of NF-κB and MAPK pathways, together with apoptosis and ferroptosis-like mechanisms, contributes to hepatocellular injury and tissue remodeling. These effects are further reinforced by gut–liver axis interactions, which facilitate the transport of inflammatory mediators and contaminants to hepatic tissue [14].

Overall, these findings support a multi-pathway model of hepatotoxicity in which MNPs act as both particulate stressors and modulators of contaminant dynamics, thereby amplifying hepatic injury under mixture exposure conditions.

### 10.4. Knowledge Gaps and Future Research Directions

Despite growing evidence of MNP–contaminant interactions, several critical knowledge gaps remain, limiting accurate risk assessment [9]. A major limitation is the lack of studies that simultaneously address both physicochemical behavior and biological effects under environmentally realistic conditions. Most experimental designs rely on pristine particles and simplified exposure scenarios.

A key unresolved issue is the relative contribution of particle-driven versus chemical-mediated toxicity [20]. Although both leaching and sorption processes are well documented, distinguishing their roles in vivo remains challenging, particularly in complex systems such as the liver, where multiple pathways converge.

Another important limitation is the insufficient characterization of desorption processes within biological systems [5]. While environmental sorption is well studied, less is known about contaminant release within the gastrointestinal tract and along the gut–liver axis. The influence of biological factors, such as enzymes, bile salts, and the microbiota, remains poorly understood.

Standardization also represents a major challenge. Variability in particle size, morphology, polymer type, concentration, and exposure duration complicates comparisons across studies [8]. Improved reporting of particle characteristics, including aging status and surface properties, is essential for reproducibility and mechanistic interpretation.

Finally, integrative and high-resolution approaches are needed to better understand mixture toxicity [40,41]. Combining transcriptomic, metabolomic, and histopathological analyses under environmentally relevant conditions will improve the translational value of zebrafish studies and enhance predictions of ecological and human health risks.

Addressing these challenges will be essential for advancing from descriptive observations toward a predictive understanding of MNP-induced hepatotoxicity in complex environmental contexts.

## 11. Modifying Factors and Methodological Considerations in MNP-Induced Hepatotoxicity

In addition to the mechanistic pathways described in Section 5, Section 6, Section 7 and Section 8, the extent and nature of micro- and nanoplastic (MNP)-induced hepatotoxicity in zebrafish are strongly influenced by physicochemical particle characteristics and experimental design parameters [8,20]. Variability across studies reflects not only differences in biological responses but also heterogeneity in particle properties, exposure conditions, and analytical approaches. Integrating these factors is essential for accurate interpretation of toxicological outcomes and for improving the ecological and translational relevance of zebrafish models.

### 11.1. Physicochemical Determinants of Toxicity

The physicochemical properties of MNPs represent primary determinants of their biological behavior and hepatotoxic potential, influencing bioavailability, cellular uptake, and intracellular interactions [7,8]. Among these, particle size, polymer composition, morphology, and surface characteristics play central roles in shaping toxicological outcomes.

Particle size is a critical factor influencing toxicity. Smaller particles, particularly nanoplastics, exhibit higher surface area-to-volume ratios and enhanced reactivity, facilitating interactions with biological membranes and translocation across epithelial barriers [5,11]. As a result, nanoplastics show greater systemic distribution and intracellular penetration, often leading to more pronounced oxidative stress, metabolic disruption, and inflammatory responses.

Polymer composition further modulates toxicity through differences in surface chemistry, hydrophobicity, and additive content [18]. While polystyrene (PS) has been extensively studied and is frequently associated with oxidative and metabolic disturbances, other polymers—including polyethylene (PE), polypropylene (PP), and polyethylene terephthalate (PET)—also induce hepatic alterations [22]. These findings suggest that many toxicological responses are driven by general particle-related mechanisms, although polymer-specific properties, such as additive composition, may influence the magnitude of effects.

A notable limitation of the current literature is the predominance of polystyrene (PS)-based model systems. In the present synthesis, approximately 50% of the included studies employed PS particles, whereas polyethylene (PE), polypropylene (PP), and polyethylene terephthalate (PET) collectively accounted for less than 20% of the studies. In addition, a substantial proportion of investigations (~30%) used mixed or insufficiently characterized micro- and nanoplastic formulations, further limiting polymer-specific interpretation. Studies employing biodegradable polymers or environmentally aged particles were comparatively rare. This distribution highlights a systematic bias toward simplified PS-based systems that do not fully reflect the physicochemical diversity of environmental MNPs, which are typically dominated by fragmented PE/PP debris and fibrous polyester materials [39]. Consequently, certain observed effects—particularly those related to cellular uptake, intracellular interactions, and potential leaching of styrene-derived compounds—may be partially polymer-specific. In contrast, core mechanisms such as oxidative stress, inflammatory signaling (e.g., NF-κB/MAPK activation), metabolic disruption, and regulated cell death pathways appear to be conserved across polymer types, although their magnitude and kinetics may vary with particle composition, morphology, and environmental conditions.

Comparative studies have also shown that alternative materials, such as biopolymer-based particles, may exhibit distinct toxicological profiles compared with conventional plastics, although adverse effects are still observed [58].

Particle morphology represents an additional determinant. Environmental microplastics typically occur as irregular fragments or fibers rather than uniform spheres, and these differences can influence surface interactions, adsorption capacity, and tissue-level effects [56]. Finally, this discrepancy contributes to variability between laboratory findings and environmental observations.

Surface modification, particularly through environmental aging and eco-corona formation, further alters particle behavior [33]. Weathering processes, such as photooxidation and biofouling, modify surface chemistry and promote adsorption of organic matter and contaminants, thereby influencing cellular uptake and toxicokinetics. Consequently, the use of pristine particles in experimental systems may not fully reflect real-world exposure conditions.

### 11.2. Biological Modifiers of Susceptibility

Host-related biological factors play a significant role in modulating susceptibility to MNP-induced hepatotoxicity [25]. Variables such as developmental stage, sex, and physiological condition can influence both the magnitude and nature of hepatic responses.

Age-dependent susceptibility has been consistently reported, with senescent zebrafish exhibiting more pronounced oxidative stress, metabolic disturbances, and histopathological alterations compared with younger individuals [25]. This increased vulnerability is likely related to reduced antioxidant capacity and impaired metabolic regulation associated with aging.

Sex-specific differences have also been described, with some studies reporting stronger metabolic and oxidative responses in females, potentially reflecting hormonal influences on energy metabolism [23]. Nutritional status further modifies susceptibility, as co-exposure to MNPs and high-fat diets exacerbates hepatic steatosis and metabolic dysregulation [42]. These findings highlight the importance of considering host physiology when interpreting toxicological outcomes.

### 11.3. Experimental Design and Exposure Considerations

The interpretation of MNP-induced hepatotoxicity is strongly influenced by experimental design, particularly exposure conditions [16]. Substantial variability in particle concentration, exposure duration, and model systems complicates direct comparison across studies.

Exposure concentration represents a key variable. While some studies employ environmentally relevant levels in the µg/L range, many use higher concentrations (mg/L) to elicit measurable effects within experimental timeframes [9]. High-dose studies are useful for identifying mechanistic pathways but may overestimate the magnitude and speed of toxic responses compared with real-world conditions.

Particle selection is another limitation. Many experimental studies rely on well-characterized spherical polystyrene particles, whereas environmental MNPs are heterogeneous in composition, size, and morphology [8]. This discrepancy reduces ecological realism and may influence observed biological responses.

Exposure duration also plays a critical role. Acute exposures typically reveal early stress responses, whereas chronic exposures provide insight into progressive alterations such as steatosis, inflammation, and tissue remodeling [4]. Integrating findings across exposure durations is therefore essential for understanding the full spectrum of hepatotoxicity.

While these conditions are useful for mechanistic exploration, they may overestimate the magnitude and rapidity of hepatotoxic responses under real-world exposure scenarios.

### 11.4. Analytical and Methodological Advances

Recent methodological advances have significantly improved the study of MNP toxicity in zebrafish [31,40]. Advanced imaging techniques, such as hyperspectral stimulated Raman scattering microscopy, enable label-free visualization of particle distribution within tissues, providing detailed insight into biodistribution patterns.

Quantitative analytical methods, including MALDI-TOF mass spectrometry, have enhanced the detection and measurement of nanoplastics in biological samples, facilitating studies of toxicokinetics and tissue retention. In parallel, omics-based approaches—such as transcriptomics, metabolomics, and lipidomics—allow comprehensive characterization of molecular responses to MNP exposure [40].

In vitro systems, including zebrafish liver cell models, further contribute to mechanistic understanding by enabling controlled investigation of cellular responses, including oxidative stress, metabolic disruption, and protein corona effects [33]. These approaches complement in vivo studies and support mechanistic interpretation.

### 11.5. Key Limitations and Future Directions

Despite substantial progress, several limitations continue to constrain the interpretation and comparability of MNP toxicology studies [16,20]. One major challenge is the insufficient characterization of particle properties, including size distribution, morphology, surface chemistry, and aging status, which limits reproducibility and cross-study comparison.

The detection and quantification of nanoplastics remain technically challenging due to their small size, resulting in an incomplete understanding of their environmental distribution and biological accumulation [9]. These analytical limitations represent a major barrier to accurate exposure assessment.

Another critical issue is the difficulty in distinguishing between particle-driven and chemical-mediated toxicity [15,20]. Because MNPs can both release intrinsic additives and adsorb external contaminants, disentangling these mechanisms in vivo remains challenging. Comparative studies using pristine particles, leachates, and environmentally conditioned materials are needed to clarify these contributions.

Standardization of experimental protocols is also essential. Variability in particle characteristics, exposure conditions, and endpoints limits comparability and hinders the development of predictive toxicological frameworks [8].

An additional limitation arises from the predominance of PS-based model systems (see Section 11.1), which may limit the ecological representativeness and polymer-specific interpretation of the observed effects.

Future research should prioritize environmentally realistic exposure scenarios, including aged particles and complex contaminant mixtures, as well as long-term experimental designs [16]. Moreover, integrating advanced analytical techniques with histopathology and multi-omics approaches will be critical to achieving a comprehensive and mechanistic understanding of MNP-induced hepatotoxicity.

## 12. Conclusions and Future Perspectives

Recent experimental studies investigating micro- and nanoplastic (MNP) exposure in combination with co-occurring environmental contaminants consistently identify the liver as a central target organ of mixed-contaminant toxicity in aquatic organisms. Accumulating evidence demonstrates that MNPs can localize within hepatic tissue following environmental exposure and induce a broad spectrum of structural and functional alterations, including hepatocellular vacuolization, steatosis, inflammatory infiltration, necrosis, and metabolic dysregulation, collectively reflecting disruption of hepatic homeostasis [4].

At the mechanistic level, oxidative stress emerges as a core driver of MNP-induced hepatotoxicity. This process is characterized by increased reactive oxygen species production, impaired antioxidant defenses, and lipid peroxidation, and is closely associated with mitochondrial dysfunction, activation of inflammatory signaling pathways such as NF-κB and MAPK, and the initiation of regulated cell death mechanisms, including apoptosis and ferroptosis [35]. In parallel, disturbances in lipid and glucose metabolism contribute to steatosis and broader metabolic imbalance [5].

Importantly, MNP-induced hepatotoxicity cannot be explained solely by particle-driven effects. Instead, the toxicological impact reflects the combined contributions of direct particle interactions and chemical-mediated mechanisms arising from the dual-origin nature of MNP exposure. Through leaching and sorption/desorption processes, MNPs can release intrinsic additives while simultaneously modulating the transport, bioavailability, and hepatic delivery of co-occurring environmental contaminants, including heavy metals, pesticides, polycyclic aromatic hydrocarbons (PAHs), per- and polyfluoroalkyl substances (PFASs), antibiotics, and algal toxins. As a result, these interactions frequently amplify oxidative stress, inflammatory responses, metabolic dysregulation, and hepatocellular injury under co-exposure conditions [16].

A further critical dimension of MNP toxicity involves systemic interactions mediated by the gut–liver axis. Disruption of intestinal barrier integrity and microbiota composition promotes the translocation of microbial products and metabolites, which subsequently influence hepatic inflammation and metabolic regulation. Within this framework, MNPs may also facilitate the transport of associated contaminants across biological barriers, reinforcing the interconnected nature of particle-driven and chemical-mediated toxicity pathways [26].

Collectively, current evidence supports a multi-pathway model of hepatotoxicity in which MNPs act both as particulate stressors and modulators of contaminant dynamics. This integrative perspective helps explain the variability—and often increased severity—of hepatic effects observed across studies, particularly under mixture exposure conditions.

From a translational perspective, zebrafish (*Danio rerio*) represents a valuable vertebrate model for investigating hepatotoxic mechanisms induced by environmental contaminants, including MNPs [1,2]. Its relevance is supported by substantial genetic, molecular, and physiological conservation with mammals, particularly in pathways governing oxidative stress, inflammation, and metabolic regulation. Furthermore, key signaling cascades implicated in MNP-induced hepatotoxicity—such as NF-κB, MAPK, and PPAR pathways—are highly conserved across vertebrate species, thereby enabling identification of shared pathological outcomes [35].

Nevertheless, important limitations must be considered when extrapolating zebrafish findings to human health risk assessment. Differences in liver organization, metabolic rate, and exposure routes may influence toxicokinetics and tissue distribution. In addition, many experimental studies use exposure concentrations that exceed environmentally relevant levels, limiting direct quantitative translation. Moreover, the predominance of polystyrene-based model particles does not fully reflect real-world exposure conditions, which are characterized by heterogeneous polymer mixtures [20].

Despite these limitations, zebrafish remains a powerful model for mechanistic toxicology, particularly due to its suitability for high-throughput screening, in vivo imaging, and integrative omics approaches [31,40]. To maximize the translational relevance of zebrafish data, future research should integrate zebrafish findings with mammalian models and human-relevant systems, such as liver organoids and primary hepatocyte cultures, to bridge the gap between experimental data and human health risk assessment.

Despite significant advances, several key knowledge gaps remain. Many studies rely on pristine particles and simplified exposure designs that do not adequately capture environmental complexity. Future research should prioritize environmentally aged particles, realistic contaminant mixtures, and long-term exposure paradigms, together with improved analytical approaches to better characterize nanoplastic distribution and distinguish between particle-driven and chemical-mediated mechanisms in vivo.

Further progress will depend on integrative experimental strategies combining histopathology with advanced molecular and omics-based approaches [40,41]. Particular attention should be given to eco-corona formation, particle aging, and contaminant interactions in shaping toxicological outcomes. Expanding comparative studies across species and exposure systems will be essential to enhance both ecological and translational relevance.

In conclusion, zebrafish represents a robust and versatile model for elucidating the complex mechanisms underlying micro- and nanoplastic-induced liver toxicity. Advancing the field will require integrated experimental frameworks that consider MNPs not only as particulate toxicants, but also as dynamic modulators of contaminant transport, bioavailability, and mixture toxicity under environmentally realistic conditions [15].

## Figures and Tables

**Table 1:** Representative zebrafish studies on single micro-/nanoplastic exposure and hepatic effects.

Polymer Type	Particle Size	Concentration	Exposure Duration	Life Stage	Key Hepatic Endpoints	Main Findings	Reference
PS	5 µm; 20 µm	~0.5–50 mg/L	7 days	Adult	Accumulation, histopathology	Size-dependent hepatic accumulation; smaller particles reach the liver	[3]
PS NPs	~50 nm	~0.1–10 mg/L	5–7 days	Embryo/larvae	Distribution, toxicity	Systemic distribution, including the liver	[11]
PS NPs	~100 nm	~1 mg/L	7–14 days	Adult	Biodistribution	Persistent accumulation in the liver	[21]
PS MPs	~5 µm	20–100 µg/L	21 days	Adult	Steatosis, lipid metabolism	Disrupted glycolipid metabolism	[4]
PS NPs	Nano-range	NR	Chronic	Adult	Metabolic alterations	Hepatic metabolic disruption	[5]
PE MPs	~10–45 µm	~100 mg/L	96 h	Adult	Oxidative stress	Acute toxicity and biochemical changes	[22]
PET fibers	Microfibers	NR	14 days	Adult	Endocrine + oxidative stress	Fiber morphology alters toxicity	[23]
PS MPs	Micro-size	~10–100 mg/L	14–21 days	Adult	Gene expression	Biological pathway disruption	[24]
PS NPs	~50 nm	~1–10 mg/L	3–7 days	Larvae	Inflammation	Hepatic immune activation	[6]
PS MPs	Environmental MPs	~1–100 µg/L	21–28 days	Senescent	Metabolomics	Age-dependent hepatotoxicity	[25]
Mixed MPs	Micro-size	~10 mg/L	7 days	Adult	Gut–liver axis	Dysbiosis + hepatic effects	[14]
PS NPs	Nano	NR	14 days	Adult	Lipid metabolism	Gut–liver metabolic disruption	[26]
PP MPs	Micro-size	~1–10 mg/L	14 days	Adult	Histopathology	Combined intestinal–hepatic injury	[27]
PGA MPs	Micro-size	NR	14–28 days	Adult	Metabolic disruption	Biodegradable plastics still toxic	[28]
PET NPs	Nano	NR	Embryonic	Embryo	Oxidative stress	Developmental + hepatic stress	[29]

PS, polystyrene; PE, polyethylene; PP, polypropylene; PET, polyethylene terephthalate; PGA, polyglycolic acid; MPs, microplastics; NPs, nanoplastics.

**Table 2:** Representative zebrafish studies on co-exposure (MNPs + contaminants).

Polymer Type	Co-Contaminant	Particle Size	Concentration	Exposure Duration	Life Stage	Key Hepatic Endpoints	Main Findings	Reference
PS MPs	Cadmium	Micro	NR	Chronic	Adult	Steatosis, metabolic dysregulation	Enhanced hepatotoxicity via gut–liver axis	[46,48]
PS MPs	Sulfamethoxazole	Micro	NR	7–14 days	Adult	ROS, MAPK activation	Synergistic oxidative damage	[17]
MPs	PFOS/F-53B	Micro	NR	Chronic	Adult	Lipid metabolism	Increased toxicity under co-exposure	[49]
PE MPs	Oxytetracycline	Micro	NR	7–14 days	Adult	Dysbiosis, liver dysfunction	Antibiotic–plastic interaction	[52]
PS NPs	Microcystin-LR	Nano	NR	7 days	Adult	Oxidative stress	Increased toxin uptake	[53]
NPs	Sodium fluoride	Nano	NR	Chronic	Adult	Lipid metabolism	Combined metabolic toxicity	[54]
PS MPs	Chlorpyrifos	Micro	NR	7–21 days	Adult	Gut microbiota, liver stress	Microbiota-mediated toxicity	[50]
MPs	Imidacloprid	Micro	NR	Chronic	Adult	Gene expression	Enhanced hepatotoxicity	[45]
MPs	Phenanthrene	Micro	NR	7–14 days	Adult	Oxidative stress	Synergistic toxicity	[51]
MPs	Cadmium	Micro	NR	Chronic	Adult	Oxidative stress	Additive toxicity	[47]
PS MPs	Microcystin-LR	Micro	NR	7–14 days	Adult	Histopathology	Increased liver injury	[43]
PS MPs	Copper/NOM	Micro	NR	Chronic	Adult	Bioaccumulation	Vector/sink effects	[56]
MPs	Cadmium	Micro	NR	Chronic	Adult	Accumulation, toxicity	Increased Cd uptake	[55]
MPs	Ben-zo[a]pyrene	Micro	NR	Trophic	Whole organism	Transfer	Food-chain transport	[10]
NPs	β-HCH	Nano	NR	Chronic	Adult	PANoptosis	Multi-pathway cell death	[57]

PS, polystyrene; PE, polyethylene; MPs, microplastics; NPs, nanoplastics; ROS, reactive oxygen species. Many studies do not consistently report exposure concentrations, further limiting comparability.

## Data Availability

No new data were created or analyzed in this study. Data sharing is not applicable to this article.
